# Protecting the Borders: Tissue-Resident Memory T Cells on the Front Line

**DOI:** 10.3389/fimmu.2015.00090

**Published:** 2015-03-03

**Authors:** Kimberly S. Schluns, Kimberly D. Klonowski

**Affiliations:** ^1^Department of Immunology, The University of Texas MD Anderson Cancer Center, Houston, TX, USA; ^2^Department of Cellular Biology, University of Georgia, Athens, GA, USA

**Keywords:** tissue-resident memory cells, T cells, mucosal tissues, migration, T cell differentiation

This research topic of Frontiers in Immunology focuses on T cells residing in mucosal tissues and is dedicated to a pioneer in the field, Leo Lefrancois. As mucosal tissues are the major portals of pathogen entry, the generation and functions of tissue-resident T cells are crucial for mediating protection and immune homeostasis at these sites. Unlike previously described T cell subsets, these tissue-resident T cells exhibit regional specificity with minimal systemic migration, most being previously activated or memory T cells. While tissue-resident memory T cells (Trm) display some overlapping phenotypes with other circulating T cell subsets, their origins and developmental pathways remain elusive.

The most extensively described tissue-resident T cells are those present in intestines, lung, and skin. While CD8 Trm derived from these mucosal tissues express core genetic profiles (1), unique patterns also exist, suggesting some tissue-specific programing *in situ*. Reviews in this issue by Shane et al. (2) and Mueller et al. (3) highlight the role of the respiratory and cutaneous microenvironment, respectively, on full commitment to the Trm lineage. Both reviews describe the cellular interactions and other regional cues that may support Trm differentiation and sublocalization (2, 3). Interestingly, dependency on cytokines and lifespan differs between these sites suggesting that not all Trm are equal. These and many other studies reviewed in this issue analyze Trm cells after tissues dissociation. Unfortunately, this approach isolates only a fraction of immune cells present *in situ* and, importantly, fails to reveal spatiotemporal cell–cell interactions. Undeniably, studies using microscopy allow one to gain a better perspective of the dynamics of T cells motility and migration patterns within the unique architecture of specific tissues. Benechet et al. (4) reviews the literature that has utilized various imaging technologies to decipher the migration activities of T cells. Future analysis of unperturbed Trm within their privileged niche will likely reveal unappreciated interactions and behaviors that improve our understanding of Trm biology.

Most studies investigating Trm focus on CD8 T cells; however, pathogen-specific CD4 Trm in mucosal tissues have also been discovered. In this issue, three reviews cover CD4 T cells starting with a global description of mucosal resident CD4 T cells presented by Turner and Farber (5). This is followed by a more focused description of the CD4 T cell responses resulting from encounters with intracellular bacteria, such as *Salmonella* and Chlamydia, which invade the intestines and female reproductive tract, respectively (6). Lastly, Gratz and Campbell (7) provide a new paradigm that includes T regulatory cells (Treg) among subsets of Trm. Indeed, Treg are enriched in the mucose to maintain a tolerogenic environment.

To understand the normal forces exerted on mucosal resident T cells, knowledge of the unique microenvironment inhabited by these T cells is essential. Despite the inherent tolerogenic nature of the mucosa, pathogen exposure and associated inflammation overrides the naturally suppressive environment, promoting Trm development. Inflammatory cytokines regulate the expression of chemokine receptors and other homing molecules to promote the migration of effector T cells to distinct locations where they will commit to the Trm lineage. Kim and Harty (8) highlight the effects of inflammatory cytokines present during different stages of the immune response on resultant CD8 T cell differentiation. Specifically, they describe an unappreciated role of IL-15, which promotes trafficking to inflamed tissues as well as the contrasting roles of TGF-β in the formation and retention of Trm. In addition to infection, commensal bacteria also likely influence Trm cells. The review by Spasova and Surh (9) describes how the gut microbiota is sensed by pattern recognition receptors such as TLR and NOD-like receptors. Subsequently, how unique immune cells populations highly represented in the intestines (ILC, specific subsets of DCs, Th17, Tregs, and IEL) interpret these signals and influence the gut microenvironment, including the persistence and functions of Trm, is also discussed. Together, these reviews draw attention to the complexity of specific microenvironments and their impact on Trm development and function.

While improving our knowledge of Trm will surely help to develop better clinical strategies to promote mucosal immunity, Sowell and Marzo (10) suggest that we should proceed with caution. In their opinion piece, they suggest that Trm may be refractory or inhibited by certain strategies, which modify conventional memory T cell responses, and vice versa. This is exemplified by inhibition of mTOR signaling that enhances memory T cell generation at the expense of effector T cell accumulation in the mucosa. As such, understanding how to specifically enhance development of Trm during vaccination will be of great value in the future.

In summary, this special issue highlights our evolving understanding of tissue-resident T cells. But many questions remain unanswered. For example, while tissue-resident T cells were originally described as a population unique to mucosal tissues, more recent studies have identified T cells with similar attributes in non-mucosal tissues, such as the brain and lymph nodes (11, 12). Does this indicate that all tissues can harbor a permanent T cell population or only those with little access to circulating immune cells? Furthermore, are specific Trm pools within a given site undergoing continued attrition and replacement with repeated infections? Filling in these knowledge gaps will be essential to expanding our understanding of Trm during pathogen infection or other mucosal perturbations. Subsequently, strategies that exploit the functional responses of tissue-resident T cells can be developed.

## Conflict of Interest Statement

The authors declare that the research was conducted in the absence of any commercial or financial relationships that could be construed as a potential conflict of interest.
